# Hospital Childbirth: Perspectives of Women and Professionals for a Positive Experience—A Qualitative Study

**DOI:** 10.3390/ijerph181910238

**Published:** 2021-09-29

**Authors:** Beatriz Pereda-Goikoetxea, Joseba Xabier Huitzi-Egilegor, Josune Zubeldia-Etxeberria, Maria Jose Uranga-Iturrioz, Maria Isabel Elorza-Puyadena

**Affiliations:** Faculty of Medicine and Nursing, Department of Nursing II, University of the Basque Country, 20014 San Sebastian, Spain; josebaxabier.huitzi@ehu.eus (J.X.H.-E.); josune.zubeldia@ehu.eus (J.Z.-E.); mariajose.uranga@ehu.eus (M.J.U.-I.); mariaisabel.elorza@ehu.eus (M.I.E.-P.)

**Keywords:** continuity of patient care, delivery, midwifery, qualitative study, women’s experiences, woman centered-care

## Abstract

The perception and interpretation of childbirth are changing as values change. This requires women and professionals to adapt to new circumstances. The objective of this study was to analyze the perspectives of women and professionals on hospital birth and to identify improvement areas in order to achieve a positive perinatal experience. A qualitative prospective study with a phenomenological approach was conducted using semi-structured interviews with women, two and eight months after childbirth, participant observation, and professional focus groups. The analysis of the transcribed texts involved a thematic inductive approach. Four improvement areas emerged from the analysis: (a) strengthening communication and the therapeutic relationship; (b) unifying criteria between hospitals and primary care centers to provide coordinated and coherent information; (c) involvement of the partner in the whole process of pregnancy-childbirth-puerperium; (d) improvement of the spaces used in prenatal care and births. The need for a continuity of care from the beginning of pregnancy to the postpartum period is emphasized, which requires an improvement in information, participation, and the promotion of shared decision-making. To this end, coordinated interdisciplinary work, involvement of the partner and the improvement of the spaces used in prenatal care and births are essential.

## 1. Introduction

Childbirth is a complex process with interrelationships between physiological and psychological processes influenced by social, environmental, organizational, and political contexts [1]. The WHO, when defining a positive birthing experience [2], emphasizes the significance of both clinical and psychological safety. The practical and the emotional support of staff with adequate skills are both necessary in order to give women a sense of achievement, control, and participation in decision-making about their care.

In the context of childbirth care, some authors consider the following aspects essential: the communication of information [3,4], participation in decision-making [3,5,6], a sense of control [7,8], and the quality of relationship with caregivers [4,9].

Aannestad et al. [10], Beecher et al. [11], Hunter et al. [12], NICE [13] and Thelin et al. [14] highlighted the importance of establishing a supportive relationship based on trust and mutual respect between women and professionals, in which the singularity of each woman is considered, and she is taken care of as a unique person. This aspect has been promoted by the WHO in its latest recommendations [2], and has been highlighted as a fundamental aspect for women to fulfil their personal and sociocultural beliefs and expectations.

In recent decades, the role assigned to women in relation to health professionals has changed. The paternalistic model has gradually been abandoned and in turn the autonomy of women has increased [15], while care has focused on “being with the woman” in the birth process. Hunter [16] defines this supportive role as the provision by professionals of emotional, physical, psychological and spiritual support, taking into account the wishes of the woman. This model of woman-centered care is also characterized by the transfer of “power” to women, and it encourages informed decision-making, so that women actively participate in care and decision-making regarding their health, while expressing their preferences and expectations [17]. Women demand the opportunity to express their views on the positive and negative aspects of their experiences [18], and this has meant, for professionals, a continuous adjustment to new circumstances [19].

No qualitative works have been found that address these abovementioned issues in Spain. In the last decades in Spain, care during and after childbirth has been promoted through the implementation of the “*Strategy for Normal Delivery Care*” [20] and the creation of the *Clinical Practice Guide on Normal Delivery Care* [21]. With all of this, an attempt has been made to achieve excellence in care in a process of great significance at the personal, family and social level, and where it is also very important for the promotion of physical, mental and emotional health of both the woman and the newborn. In fact, the quality of care at delivery and birth not only conditions morbidity and mortality rates, but also other aspects such as dissatisfaction with the experience, loss of self-esteem, depression, feelings of incompetence, difficulties in bonding with the newborn, difficulties in breastfeeding, and problems with upbringing [22].

To obtain an overall perspective of births and maternity care, it is necessary to listen to both professionals and women. This will allow professionals to identify the areas which should be improved in order to make it a positive experience. The aim of this work is to analyze the perspectives of women and professionals on hospital birth, and to identify improvement areas in order to achieve a positive perinatal experience.

## 2. Materials and Methods

This study was conducted in the labor ward of the Donostia University Hospital, a public tertiary care referral hospital in Gipuzkoa, Spain, where 3674 babies were born in 2016 and 3517 in 2017.

This study used a prospective qualitative method with an interpretive phenomenological approach [23] to understand childbirth from the “inside,” that is, from the subjective experience of women who had given birth and the professionals who had attended them.

The sample was extracted from women who came to the Donostia University Hospital to give birth from 1 January to 31 May 2016 and from professionals (obstetricians, nursing assistants, and midwives) who worked at the labor ward of the hospital in 2016 and 2017.

The inclusion criteria were as follows:

- Women who gave birth to a live newborn, gestation greater than or equal to 37 weeks, cephalic presentation, 18 years of age or older, with adequate oral and written comprehension of Spanish and/or Basque language, and competent to understand and provide written informed consent.
- Professionals who had worked for at least 2 years in the labor ward.

Women in the following circumstances were excluded from the study: twin gestation; scheduled caesarean; admission of the newborn or the mother to the intensive care unit; women suffering from a psychiatric illness, and women who had been attended by the main researcher. Scheduled caesarean sections were excluded because the professionals who cared for these women were not obstetric specialists. In the case of professionals, in order to include a wide range of opinions, none was excluded.

The main researcher recruited the women in the puerperium unit and the professionals in the labor ward. The recruitment of women participants began with convenience sampling, followed by theoretical sampling after interviewing 19 women. The variables identified in the strategic selection of the cases were: the type of start and end of labor, the use of analgesia, the state of the newborn and the parity. For the professionals, recruitment began with intentional sampling and then snowball sampling.

The resulting final sample size was as follows: 42 women for the interview 8 weeks after childbirth; 32 women for the interview 8 months after childbirth, and 15 health professionals. Table 1 and Table 2 show different characteristics of the women and the professionals who participated in the study.

In the case of the 51 women recruited at the hospital, 9 did not want to participate in the first interview, claiming they did not have time. Thus, the sample was reduced to 42 women. Later, 10 women did not participate in the second interview. The reasons for not participating in the second interview were the following: inability to contact them (5 cases), they did not attend the appointment (1 case), and they did not have time (4 cases). It was decided to stop the progressive incorporation of informants when the information collected was repeated and did not add anything relevant to what was already known, that is, when the saturation principle was reached [24].

All the participants were informed orally and in writing about the study’s purpose by the main researcher. After their acceptance, the informed consent form was provided and explained to them.

The data collection techniques were as follows:

- Individual semi-structured interviews were conducted 8 weeks (M-A) and 8 months (M-B) after childbirth. An open question began both the first and second interviews: at the 8th week, the question was “*How was your birthing experience?*”; at the 8th month, the question was “*What do you remember about your birthing experience?*”. During the interviews, the women were free to describe their experiences [25]. The interview duration ranged from 20 to 65 min, and they were conducted in the place chosen by the woman: at home, in cafeterias or in parks.
- Focus groups: The 4 focus groups (G1, G2, G3, and G4) included obstetricians, nursing assistants, and midwifery personnel who worked at the labor room. Three homogeneous groups and one heterogeneous group in terms of discipline were formed (see Table 2). The meetings were held in a hospital room, respecting the wishes of the participants. To guide the sessions, the following question was asked: “*How do you view women’s childbirth experiences?*” During the process, equitable participation was encouraged. At the end of the sessions, emerging topics and subtopics were discussed. Finally, a summary was made aloud and participants were encouraged to add or rectify the data.

Both the semi-structured interviews with the women and with the focus groups of professionals were conducted by the main researcher. In both cases, oral consent was requested to audio record using a digital recorder.

- Participants’ observation: Participant observation was conducted in “natural” field situations [26] and it served to obtain direct experience of the childbirth phenomenon. The data obtained in situ were compared with the information obtained in the interviews and focus groups.
- Field journal: This was used to deepen in the meaning of the discourses and behaviors.

The data were analyzed jointly and thematically following the guidelines proposed by Braun and Clarke [27]. Several phases were differentiated: familiarization with literal transcriptions; segmentation of the text into units of meaning; identification and coding of units of meaning (a total of 260 codes emerged); continuous comparison; definition of categories and grouping into broader themes by creating semantic networks and matrices [28]; sharing of the results, and description of emerging issues through a final report.

**Figure 1:**
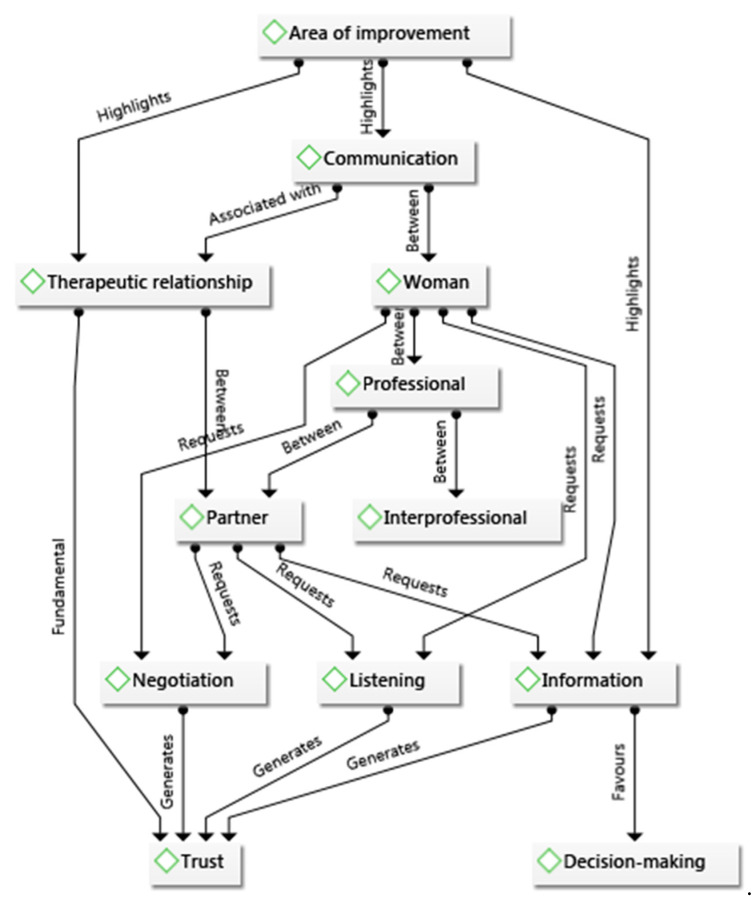
Creation of the semantic network in the analysis process.

The approximation was inductive, circular, with an attitude of reflection and constant feedback [29], giving meaning to the whole data set. Examples of the analysis process are shown in Table 3 and Figure 1.

ATLAS.ti 7 software was used to manage, identify, organize, analyze, and communicate the set of topics representing the content of the texts in the research [27].

The criteria of Lincoln and Guba [30], credibility, transferability, confirmability and dependability, ensured the reliability of the findings.

To ensure credibility, a triangulation of the data, methods, and analyses was carried out, combined with continuous reflection based on memoranda and field notes.

Transferability was ensured by the detailed description of the participants’ discourses and the methodology. To promote and reinforce the transparency of the analysis, literal extracts were used that described the reality of the experience of childbirth and care.

Confirmability and dependability: The principal investigator conducted all the interviews and focus group discussions to ensure maximum coherence. The results were compared with the existing literature, with the final confirmation of the participants, and the support of an audit follow-up conducted by the other researchers. The final report of the consensual results was sent to the participants. The COREQ checklist was used as a guide for this study [31].

The study was approved by the Clinical Research Ethics Committee of the Health Area of Guipuzcoa (reference: BPG-APH-2015-01; 21 July 2015). Participation was free and voluntary. The confidentiality of the information and the anonymity of the participants were ensured at all times through the assignment of codes. After the completion of the study, all the participants received feedback on the results.

## 3. Results

The reflections and descriptions provided by the women and the professionals offered a comprehensive view of the multiple components of the hospital delivery experience.

**Figure 2:**
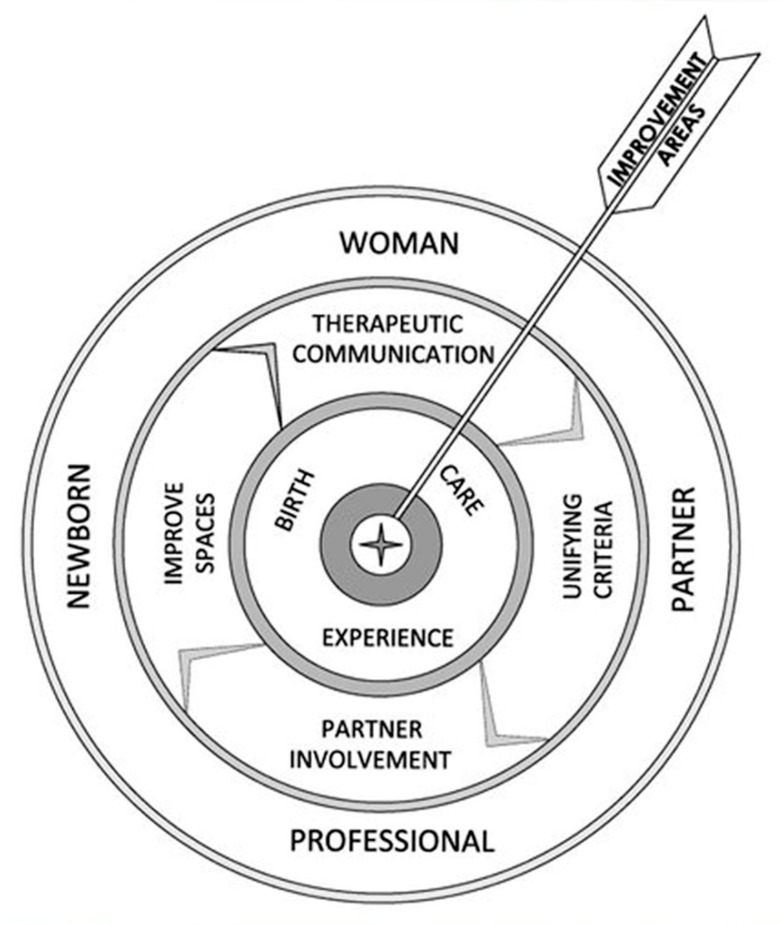
Representation of the areas for improvement raised by the participants.

The themes that emerged when describing the experience of childbirth and care constitute the areas for improvement proposed by the participants, and have been represented in Figure 2. This figure shows: in the outer layer, the participants of the hospital delivery experience; in the intermediate layer, the areas of improvement described by the participants, and in the inner layer, the processes in which said improvements have to be applied. The three layers interrelate with each other for a positive hospital birth experience.

The main areas for improvement that emerged from their comments were the following: (a) strengthening communication and the therapeutic relationship; (b) unifying criteria between hospitals and primary care centers to provide coordinated and coherent information; (c) involvement of the partner in the whole process of pregnancy-childbirth-puerperium; (d) improvement of the spaces used in prenatal care and births.

### 3.1. Strengthening Communication and the Therapeutic Relationship

The need to improve communication between all parties involved was expressed in order to foster responsible decision-making and facilitate coping with habitual or stressful situations.

The women requested clearer and more truthful information about what was happening to them at each stage and about the different coping strategies available.

*“An explanation: this is what’s happened, we’ve done this, we’ve done the other… From now on you are going to notice this. This is normal, this is not normal”*

(25 M-A, 49:10).

The professionals identified the need to develop communication skills.

*“Sometimes we tell them too many things, and then we cannot get into detail and that is when they are left with biased information”*

4 G-1 (155:4).

To establish bonds that build trust and stimulate the sharing of emotions, it was considered essential to propitiate and receive information with an empathetic, cordial, and respectful attitude during the practice of care.

*“It would be better to try to say it another way in that moment of so much pain and when they are not well… a little more tactfully”*

17 M-A (34:32).

*“Any suggestions? They are very good professionals, but there are some who should change their attitude”*

23 M-A (45:63).

The participants proposed maintaining a personalized therapeutic relationship in which, in addition to focusing on the task, the professionals did not neglect the patient’s emotional circumstances. They considered it necessary that, in addition to addressing the bio-physiological aspects of the situation, importance be given to the professional–woman interaction through open dialogue, active listening, sharing of individual singularities and negotiation in decision-making.

The professionals agreed with the issues raised by the women, and stressed the need to improve the methods and timing of providing information. Postpartum support was proposed to resolve any remaining doubts.

*“I feel like we may be lacking reassurance afterwards. For example, an instrument in a situation of urgency… they start wondering: is it because I have not pushed? Why have they induced me? Why?… These doubts remain unsolved”*

1 G-3 (149:20).

### 3.2. Unifying Criteria between Hospitals and Primary Care Centers to Provide Coordinated and Coherent Information

The need to unify criteria between the hospital and primary care was raised.

The women thought that sometimes they received contradictory information, which generated doubts and made it difficult for them to make decisions autonomously. That is why they demanded a unification of criteria between primary care centers and the hospital.

The disparity of criteria and contradictory information was observed especially in relation to breastfeeding and postpartum recovery.

*“Everyone is willing to help you. What happens is that there are 50,000 different midwives, each of whom has an opinion”*

15 M-A (30:68).

*“The different criteria should be unified. Otherwise, you feel a little confused”*

37 M-B (136:35).

The hospital professionals highlighted the need to develop information protocols agreed on between primary and hospital care, since, in their opinion, women received highly theoretical and idealized information on childbirth in primary care centers.

*“I see women sometimes come with very idealized information about childbirth; that is dangerous”*

(3G-2, 153:8).

*“I think there has been a lot of information given by theorists who have never been to a delivery room. So they are creating expectations… which are not real”*

(3G-1, 153:7).

*“In the same way that we write medical protocols, I believe that follow-up protocols must be established, information that is not biased”*

(1G-1, 149:32).

The professionals highlighted the need to strengthen teamwork and interdisciplinary communication.

*“It would be necessary to analyze: why is there a lack of communication between primary and hospital midwives? It would be important”*

(3G-1, 153:6).

*“It is important that there be more communication between gynaecologists, midwives, assistants… this teamwork does not exist nowadays”*

(3G-1, 153:21).

*“The population… has changed a lot; I believe that coordination between the hospital and the primary care centers is still pending”*

(1 G-1, 149:28).

### 3.3. Involvement of the Partner in the Whole Process of Pregnancy-Childbirth-Puerperium

All women participants agreed that involving their partner in the birth process helps them to adjust to their new role within the family. The women requested the involvement of the partner not only at the time of birth, but also during pregnancy and child-raising. To achieve this, they suggested that the partners attend antenatal classes, so that they could acquire the necessary knowledge and skills.

The joint experience would provide psychological support and increase awareness of capability and active participation in the partners.

*“That the father took part. If he is involved in the pregnancy, in childbirth, I believe that will help him be involved in the upbringing”*

(33 M-B, 133:7).

The women asked the professionals to engage the partners so that the partners feel helpful, valued and informed. By acquiring knowledge, skills and attitudes, a more active, positive and empowered experience would be favored. To this aim, they demanded that the public health service offer greater flexibility in the schedule of the courses.

*“I would like the schedule to be more flexible because, for example, my husband could not come to public antenatal classes and neither could I until I took the leave, I couldn’t go because the schedules coincided”*

(25 MA, 49:47).

They also expressed the desire for the antenatal classes to be more practical, using methodologies and didactic resources that should go beyond the simple theoretical class offered, proposing techniques for the development of both physical skills and the management of feelings and emotions.

*“Yes, I would have liked them to be more practical, they seemed very theoretical to me. We were there for two hours and the midwife would spend two hours talking and we would not speak”*

(21 M-A, 41:54).

*“Not dedicating so much time to theory, but having more practical lessons instead”*

(23 M-B, 115:5).

They also proposed that maternity leave be extended and that paternity leave be as long as maternity leave, all to ensure the care of the child, facilitate the establishment of ties, and promote joint responsibility of partners.

*“They tell you that it is advisable to breastfeed for up to six months, but your maternity leave lasts four months… and many times you cannot… there are many contradictions. Also in the case of fathers, because for me it is very important that both parents are with the child so that bonding occurs”*

(37 M-A, 73:73).

### 3.4. Improvement of the Spaces Used in Prenatal Care and Births

The need to improve some of the spaces used in primary care and in the hospital delivery service was underlined.

In primary care, the women expressed the need for the spaces in which the antenatal classes are given to be larger, so as to allow the participation of partners as well.

*“The room was very small and we all went without partners because we could not fit and we could not do the exercises”*

(28 M-B, 125:36).

In relation to hospital spaces, the women described the delivery room as a cold space, likening it to an operating room. They said that they would have appreciated a more welcoming environment, more like being at home.

*“Another thing that could change would be the space of the delivery room, which is not friendly at all”*

2 G-2 (151:59).

Finally, they also demanded pleasant spaces that encourage a respite and bonding between the woman, her partner and the newborn. A space that would allow intimacy and thus sharing of the perinatal experience was suggested.

*“That everyone have the option of being in a comfortable and intimate place with their newborn […]”*

(4 G-3, 155:34).

*“Everyone should have the option of being with their newborn”*

4 G-3 (155:34).

## 4. Discussion

The first improvement proposal highlighted in this study was strengthening communication and the therapeutic relationship. In this sense, O’Brien et al. [4] pointed out that informed choice is the basis for feeling empowered.

Both women and professionals also considered that in order to make shared decisions, in addition to information, dialogue is required. This is something that Karlström et al. [32] had already pointed out: the decisions to be made depend on the quality of the relationship established between the woman and the professional. Aannestad et al. [10] and Borrelli et al. [33] emphasized the importance of establishing a two-way relationship of trust between the woman and the professional, concluding that the feeling of trust can influence the progression of labor. Likewise, the WHO identifies effective communication among its recommendations on care during childbirth in order to achieve a positive birth experience [2], and this study adds to this recommendation the need to improve the ways and times of providing information. In addition, the WHO recommendations refer to care during childbirth and immediate care of the woman and the newborn after delivery [2]; however, the participants in this study considered care in the puerperium as an integral part of the hospital delivery experience.

The second proposal for improvement made by the women and the professionals who participated in this study was unifying the performance criteria between primary care and hospital professionals. The need to provide contextualized and coordinated information was highlighted so that women could make sense of all the information received. This would prevent women from having an idealized image of childbirth, which could interfere when making decisions. To do this, they raised the need to establish information protocols agreed on between primary and hospital care. It can be considered that this improvement proposal is novel, as it points to an aspect that should be improved in Spanish assistance. Lohmann et al. [34] highlighted the need to reach a common understanding among professionals about the essential aspects of care through more precise and standardized communication, but they do not mention the need for writing information protocols, that is, standardized information that helps minimize the gap between expectations and reality.

Another element repeated by the participants was continuity. Like Aannestad et al. [10], Bringedal and Aune [3], and Iida et al. [9], women stressed that the continuity of the professional, the team and the care environment is a key concept in the context of a positive birth experience, as this is associated with quality in relationships and the opportunity to provide care in a more comprehensive way. In the present study, a new aspect was associated with the concept of continuity: the need to work as a team throughout the pregnancy-childbirth-puerperium. According to the participants, poor teamwork results in a situation of discontinuity that could lead to a potential loss of information with an impact on the quality and safety of care.

The third need detected by the women in this study was to involve their partner more. The women considered the presence of their partner as an important source of emotional support. These three concepts were repeatedly demanded: involvement, participation and presence. This coincides with what was described by Eggermont et al. [35], who associated participation and joint experience (woman-partner) during pregnancy, childbirth and parenting with a greater emotional bond and sense of co-responsibility in caring for the child.

However, the professionals, unlike the women, barely mentioned partners, and at no time did they recognize the need for them to share the perinatal experience with the women. These divergent views sometimes led the partners to feel excluded, becoming what Steen et al. [36] described as “not patient, not visitor”. Thus, this seems to be a point to improve on for professionals, as identified in the latest WHO recommendations [2].

The fourth improvement proposal that emerged from this study was to improve the spaces used in the care of pregnancies and deliveries. The women and the professionals highlighted the importance of considering the physical environment in maternity care, coinciding with authors such as Setola et al. [37], who highlighted the influence that the physical characteristics of these spaces may have on behaviors, experiences, practices, and health outcomes at birth.

The women in the study demanded warm spaces that invited recollection where they could remain intimate and share the perinatal experience. These results coincide with those of Mondy et al. [38], who identified how the characteristics of domesticity within the birth setting may shape the experience of women in labor.

Hospital labor rooms are often associated with impersonal and functional spaces where the lack of domesticity causes women to interact with the space passively by assuming the role of patients. This research adds to previous studies the demand from both women and professionals for spaces that promote a more participatory environment for everyone involved.

Authors such as Hammond et al. [39] also emphasized aspects of spaces such as friendliness to reduce women’s stress, functionality to better meet women’s needs, and freedom to allow midwifery practice to be more flexible and responsive. However, recent studies such as that of Nilsson et al. [40] concluded that the evidence on how the characteristics of spaces affect labor and childbirth outcomes is incomplete. Therefore, there are various authors [37,40] who stress the need to carry out more research in this field in order to improve the well-being and safety of families.

### Strengths and Limitations

A significant strength of this study is that the perspectives of women and professionals on hospital childbirth were merged. In addition, this research contributes to the progress of scientific knowledge because it identifies areas of improvement for a positive perinatal experience, which may be applicable in the formulation of policies that pursue excellence in healthcare.

The fact that all the participants in this study were recruited from the same hospital could be a questionable limitation in transferring the study conclusions to other hospitals.

In any case, in order to make this transfer more viable, one of the criteria used was to exclude those cases that are only cared for in the best equipped hospitals, such as newborns admitted to intensive care. Likewise, in the sampling strategy, adequate oral and written comprehension of the official languages was established as an inclusion criterion in order to avoid misunderstandings. However, language issues should be considered when providing care, given the relevance that communication has had on the results obtained.

Another limitation could be the potential influence of the main researcher, a midwife, in the interpretation of the data, due to her own preconceptions and thoughts. To avoid it, the results were subjected to reflection and discussion in the group, until a consensus was reached on the emerging discourse.

As a continuation of this work, it would be interesting to conduct research that brings together the perspectives of professionals from both the hospital setting and primary care centers.

## 5. Conclusions

The results of this study provide areas for improvement regarding hospital delivery. They are proposals that have to do with improving communication and the therapeutic relationship, unifying action criteria between primary care centers and hospitals, involving the couple more in the whole pregnancy-childbirth-puerperium process, and improving the spaces used in maternity care.

The four areas for improvement outlined in this study are linked to achieving a positive perinatal experience.

## Figures and Tables

**Table 1:** Sociodemographic–obstetric characteristics of the women’s sample.

Characteristics		N = 42 N (%) or Mean ± Standard Deviation
Age	Age range in years: 25–43	34.6 ± 3.375
Education	Primary	4 (9.5%)
	Secondary	4 (9.5%)
	Post-secondary non-tertiary education	9 (21.4%)
	Tertiary education or higher	25 (59.5%)
Weeks of pregnancy	Range in weeks: 37–42	39.8 ± 1.313
Parity	Primiparous	25 (59.5%)
	Multiparous	17 (40.5%)
Type of delivery onset	Spontaneous	24 (57.2%)
	Induced	18 (42.8%)
Mode of birth	Normal	27 (64.3%)
	Forceps	3 (7.1%)
	Vacuum extractor	5 (11.9%)
	Spatula	2 (4.8%)
	Urgent caesarean	5 (11.9%)
Analgesia use	None	2 (4.8%)
	Local anaesthesia	3 (7.1%)
	Epidural anaesthesia	37 (88.1%)
Infant hospitalization	No	38 (90.5%)
	Yes	4 (9.5%)

**Table 2:** Characteristics of the sample of professionals in the focus groups.

Focus Groups	Age	Profession	Years in the Labor Room
G1	54	Obstetrician	24
G1	32	Obstetrician	3
G1	44	Obstetrician	19
G1	53	Obstetrician	22
G2	58	Midwife	35
G2	43	Midwife	10
G2	53	Midwife	18
G2	35	Midwife	6
G3	55	Midwife	30
G3	45	Midwife	21
G4	58	Midwife	32
G4	40	Nursing assistant	2
G4	31	Obstetrician	3
G4	50	Nursing assistant	8
G4	61	Midwife	36

**Table 3:** Description of analysis process.

	Examples of Meaning Units	Codes	Category	Theme
8 weeks	“*She was a little rude and did not talk to me, she talked to my husband. In addition, I thought, but the patient is me*” 37 M-A (136:9).	C: Addressing the patient C: Ways of giving information C: Listening C: Communication	Professional treatment and its characteristics	Strengthening communication and the therapeutic relationship
8 months	“*Suddenly, a woman who never identified herself as the midwife who was going to deliver me came in, so I was lost because I had never seen her before*” 14 M-B (28:24).	RP: Impersonal treatment RP: Cold and distant		

## Data Availability

The data presented in this study are available on request from the corresponding author. The data are not publicly available due to personal data protection.
